# Complaints: Mechanisms for prisoner participation?

**DOI:** 10.1177/14773708221094271

**Published:** 2022-06-06

**Authors:** Rebecca Banwell-Moore, Philippa Tomczak

**Affiliations:** 6123 University of Nottingham, UK

**Keywords:** prisoner participation, complaints, legitimacy, procedural fairness, dignity, agency

## Abstract

In prisons, participatory mechanisms can foster important outcomes including fairness, legitimacy and dignity. Complaints are one significant (symbolic) mechanism facilitating prisoner participation. Ombud institutions/Ombudsmen handle complaints externally, providing unelected accountability mechanisms and overseeing prisons around the world. A fair complaints process can stimulate prisoner voice, agency and rights protection, potentially averting self-harm and violence, and facilitating systemic improvements. However, complaints mechanisms are little studied. Addressing this gap, we: i) contextualise discussion by demonstrating that prisoners’ actions have directly shaped complaints mechanisms available today; ii) outline prison complaints mechanisms in the case study jurisdiction of England and Wales; and iii) provide a critical review of literature to assess whether prison complaints systems are, in practice, participatory, inclusive and fair? We conclude that complaints mechanisms hold clear potential to enhance prison legitimacy, facilitate prisoner engagement and agency, and improve wellbeing and safety. However, myriad barriers prevent prisoners from participating in complaints processes, including culture, fear, accessibility, timeliness, emotional repression, and bureaucracy. The process of complaining and experiences of these barriers are uneven across different groups of prisoners. Our article provides a springboard for future empirical research.

## Introduction

Participation is considered central to realising more democratic, sustainable and responsive public services (Bovaird, 2007), and can foster important outcomes including fairness, legitimacy and dignity within the criminal justice system and amongst individuals detained in custody (Skinns et al., 2020; Tyler, 2006). In law, meaningful participation requires adequate and timely information and an opportunity to be heard, forming a prerequisite for the legitimate authority of action, whilst denials of the right to participation inflict ‘moral harm’ and injustice (Solum, 2004: 299). In political theory, democratic optimist perspectives emphasise the hope and possibility of participatory democracy/rule by participation directing state institutions towards greater democracy and the public good (Dzur, 2012). Social scientists note that citizens around the world are demanding more participation and greater power (Cichowski, 2006). Arnstein's (2019) ‘ladder of citizen participation’ illustrates that participation ranges from tokenistic empty rituals for example being informed, consulted and placated up to legitimate forms of participation which devolve power through partnership, delegation and citizen control. Legitimate participation enables ‘the have-nots [to] join in determining how information is shared, goals and policies are set […] and can induce significant social reform’ (Arnstein, 2019: 24). In this article, we examine complaints processes as a (potential) mechanism for prisoner participation.

Prisons are closed systems of power relations (Sparks and Bottoms, 1995) where every aspect of life is heavily regulated and prisoners are dependent on staff for fulfilment of their needs. Where government has exceptional authority, rights protections ‘must be a core preoccupation’ (Sapers and Zinger, 2010: 1512). Indeed, European Prison Rule 50 requires that prisoners ‘be allowed to discuss matters relating to the general conditions of imprisonment with prison administrations and […] encouraged to communicate with the prison authorities about these matters’ (Council of Europe, 2006: 23). Some countries have enshrined prisoner participation in general prison management within primary legislation (e.g., Belgium, Germany, and Spain) (Bishop, 2006). Nevertheless, little scholarship examines how prisoners (do not) participate through mechanisms that claim to protect their rights and citizenship (Piacentini and Katz, 2017). Formal participatory mechanisms allow prisoners to express their views on issues of relevance for their collective life (Bishop, 2006). Examples of formal participatory mechanisms include legal challenges, complaints, prison councils and lived experience networks (often run by voluntary/non-profit organisations). Complaints are one significant (symbolic) mechanism facilitating prisoner participation; providing prisoners with a potential ‘lever by which to shift conditions’ (Mika and Thomas, 1988: 57).

Mechanisms for prisoner participation do not simply emerge. Examining participatory mechanisms without considering reasons for their introduction and (lack of) usage reproduces the myth of rights: that all victims are assured of their day in court and judicially/ bureaucratically affirmed rights are self-implementing social justice instruments (Scheingold, 1974). In fact, prisoners’ actions have directly shaped complaints processes available today as some Ombud institutions were established in response to prisoner riots. The establishment of Canada's *Office of the Correctional Investigator* (Federal Prison Ombudsman) in 1973 resulted from the exceptionally ‘bloody’ riots at Kingston Penitentiary in 1971 and subsequent staff retaliations towards rioters transferred to Millhaven Penitentiary (Sapers and Zinger, 2010: 1517–8). In England and Wales, concerns were expressed throughout the 1980s that mechanisms for dealing with prisoners’ complaints were inadequate, to little avail (Fowles, 1989). In April 1990, the severest prison riots in English and Welsh history started at HMP Strangeways (PRT, 1991). The subsequent Woolf Report (1991) identified frustration as a core contributor to these riots: prisoners were frustrated that their complaints (regardless of triviality) were not adequately addressed. Woolf (1991: paras 14.345–14.347) recommended a complaint procedure with final access to an Independent Adjudicator that ‘would give the whole system […] validity [and] act as a spur […] to maintain proper standards’. Accordingly, in 1994, the *Prisons Ombudsman* was established, becoming the *Prisons and Probation Ombudsman* (PPO) in 2001 (Carl, 2013). The PPO provide external complaint oversight once prisoners exhaust internal avenues.

Complaining can enable prisoners to stimulate accountability for individual failings and potentially (re)shape conditions, as investigations may initiate systemic improvement (Gill, 2020). Sapers and Zinger, from the Office of the Correctional Investigator of Canada (2010: 1515) argue: ‘through investigating individual cases, ombudsmen may highlight weaknesses […] Discovering these weaknesses is of advantage […] because […] resulting improvements in the system provide a generalized benefit’. Regarding England and Wales, former prisoner Cattermole (2019: 46) states: ‘if you are a literate person banged up with all the time in the world then it's imperative that you stand up and fight The Battle of a Thousand Forms to prove they can’t walk all over you […] [or] anyone else in the future, even if it sometimes does feel futile’. A fair, inclusive and effective complaints process is considered integral to stable and secure prisons (Prison Reform Trust (PRT) and Zahid Mubarek Trust (ZMT), 2017; Sparks and Bottoms, 1995). Participation through complaint can facilitate voice, fairness, legitimacy, dignity and wellbeing (Jackson et al., 2010; Skinns et al., 2020) potentially providing a safety valve to release frustration, which can avert self-harm, suicide, unrest, violence and riots (Ministry of Justice (MoJ) and HM Prison and Probation Service (HMPPS), 2021; Woltz, 2020).

However, (participatory) complaints mechanisms have not received scholarly attention befitting their importance (Hertogh and Kirkham, 2018). Much literature does ‘no more than call for the introduction’ of prison ombud institutions (Carl, 2013: 4). Addressing this gap, we examine the case study of prisoner complaints in England and Wales. We collate secondary data indicating how prisoners (do not) participate through this complaints process and explore the extent to which prison complaints systems are, in practice, participatory, inclusive and fair. We examine the theoretical foundations for prisoner participation and then assess complaints’ potential to facilitate participation, dignity and legitimacy. We conclude that complaints cannot be seen to represent prisoners’ concerns. Culture, timeliness, emotional repression, accessibility and demographic differences constrain prisoners’ abilities to complain. Grey literature indicated that prisoner experiences of complaints processes as ‘(un)just’ vary with demographic differences. For example, Her Majesty’s Inspectorate of Prisons (HMIP) (2020: 120) found that black and minority ethnic prisoners are significantly less likely than their white counterparts to feel that ‘complaints are usually dealt with fairly’. This requires particular consideration as disproportionate outcomes and differential treatment is problematic (Lammy, 2017).

## Theoretical foundations

Prisoner participation can foster important outcomes such as fairness, legitimacy and dignity. Fairness (or procedural justice) is ‘the fairness of procedures’ through which ‘authorities exercise their authority’ (Tyler and Huo, 2002: 135; 214). Fairness includes voice, neutrality, respect and trust, and is achieved when processes, treatment and outcomes by and from those in positions of authority are perceived to be fair and respectful (Tyler, 2006). People feel that they have been treated with procedural fairness when they are: a) given opportunity to have a *voice*: to present their side of the story, be heard and involved in decision-making; b) governed by rules *neutrally* and consistently applied; c) treated with dignity and *respect* for their rights (Jackson et al., 2010: 10); d) under *trust*worthy authorities who display unbiased decision making (Tyler and Degoey, 1996: 342). In particular, voice can facilitate inclusivity, agency and empowerment, enabling individuals to actively engage in systems to stimulate change (O’Mahoney and Doak, 2017; Weaver, 2018).

Perceptions of fairness in processes, treatment and outcomes are key to securing compliance with any system of power relations (Tyler, 2006). Establishing and maintaining legitimacy (i.e., justified authority) involves legal, political and social dimensions that are crucial to achieving social order (Beetham, 1991). Prisoners must perceive fairness for prisons to operate: for prisoners to accept and conform to the regime and for staff to have authority to implement and enforce prison rules (Jackson, et al., 2010; Tyler, 2006). Prison legitimacy is challenged by routine encounters, procedural interactions and interpersonal interactions (Sparks and Bottoms, 1995). When prisoners do not see the prison and its staff as fair, rules may be breached and prisoners’ consent withdrawn, potentially resulting in riot (Seneviratne, 2012). Procedural fairness and legitimacy can reduce distress, misconduct and violence and improve mental health (Beijersbergen et al., 2015, MoJ and HMPPS, 2021).

In police custody scholarship, Skinns et al. (2020: 1683) argue that ‘aspects of procedural justice […] may in fact be about different dimensions of dignity’. *Dignity* has potential to ‘motivate and mobilise’ citizens and criminal justice actors to bring about reform (Simon, 2017: 276), holding capacity to ‘transform the lives of individuals, particularly in situationally and structurally unequal contexts’ and to ‘transform criminal justice organisations’ (Skinns et al., 2020: 1683). Dignity reflects the right to participation (Solum, 2004) and includes three dimensions: opportunities for autonomy, the equal worth of human beings, and public decency (Skinns et al., 2020).

Loss of autonomy is a recognised ‘pain of imprisonment’ (Sykes, 1958), which can be mitigated by ‘limiting helplessness, coercion […] and dependence on staff’ (Skinns et al., 2020: 17). Opportunities to assert autonomy include having some say and playing some part in decision-making regarding prison issues (Solum, 2004). Therefore, complaints processes are potentially very important participatory mechanisms that provide a ‘legitimate outlet for grievances’ (Seneviratne, 2012: 340) and enable prisoners to: ‘challenge the conditions of their captivity’, resist ‘abusive’ policies and practices, and curtail ‘abuses of state power’ (Mika and Thomas, 1988: 56). Equal worth and public decency are demonstrated through prisoner treatment, including social relations, material conditions and a welfare-oriented culture (Skinns et al., 2020). Prisoners’ feelings of worth and decency depend on social relations and material conditions within prisons (Hulley et al., 2011). Being treated with dignity and respect ‘is consistently one of the most important issues that concern people when they are dealing with authorities’ (Jackson et al., 2010: 10). Positive, respectful and supportive relations between staff and prisoners are ‘key’ to securing control, order and safety (Beijersbergen et al., 2015, MoJ and National Offender Management Service (NOMS), 2008: 1, 41). As such, ‘complaints procedures are important mechanisms for […] supporting good prisoner-staff relationships’ (van der Valk and Rogan, 2020: 801). Moreover, positive relations with staff and decent conditions are protective factors to risk of self-harm and suicide (Ludlow et al., 2015). Material conditions (e.g., architecture, artefacts and furnishings) also contribute to wellbeing (Jewkes, 2018; Skinns et al., 2020) and have ‘potential to shape interactions and to communicate something about the manner’ in which prisoners are seen (Carr et al., 2015: 182). In prison, public decency involves prisoners being treated with respect and decency, rather than derision or degradation (Hulley et al., 2011), for example where prisoners have health care and high quality material conditions and staff laugh with, not at, prisoners (Skinns et al., 2020).

## Complaints

Complaints are enshrined within Human Rights law (van der Valk and Rogan, 2020) and perhaps amongst the most widely available mechanisms for prisoner participation. Specialised Ombud institutions increasingly provide unelected accountability mechanisms and oversee prisons globally, often being considered ‘one of the most effective models of external oversight to address prisoners’ complaints and grievances’ through unbiased, timely investigations (Sapers and Zinger, 2010: 6; see also Carl, 2013). Examples include Brazil's *Ouvidoria Nacional dos Serviços Penais*, Canada's *Office of the Correctional Investigator* (Federal Prison Ombudsman) and England and Wales’ *Prisons and Probation Ombudsman.* Whilst our analysis from England and Wales has relevance across jurisdictions, it must be extrapolated with caution. Complaint processes vary between jurisdictions. For example, in California, USA, federal law requires that prisoners go through three levels of the California Department of Corrections and Rehabilitation's internal complaints system before filing a lawsuit (Calavita and Jenness, 2013). However, in England and Wales, internal complaints can be complementary to or separate from legal action. Moreover, rates and conditions of imprisonment vary significantly between jurisdictions. England and Wales have a high rate of imprisonment within Western Europe and have had substantial staff cuts since 2012 (Sturge, 2021).

Ombud institutions rarely act on their own.^1^ Investigations operate through the participation of people (as prisoners must bring cases) and are mediated by social relationships (which can stimulate or discourage complaint) and material environments (e.g., the existence of complaint forms and prison conditions) (Tomczak, 2021). To some extent, the very (material existence of) a complaints process contributes to dignity, with for example, complaints forms and boxes symbolising voice and dignity. Complaining potentially enables prisoners to exercise agency within the disempowering prison, to tell their story, ‘get something off their chests’ (Calavita and Jenness, 2013: 71), ‘assert or protect their rights’ and maintain dignity (Behan and Kirkham, 2016: 432). Complaints processes have a ‘fundamentally important role in easing tensions and allowing people to feel that they are being treated justly’ (Day et al., 2015: 32), and in theory, complaints processes do facilitate voice, fairness and legitimacy (Weaver, 2018). But, *to what extent are prison complaints systems, in practice, participatory, inclusive and fair*?

Opportunities to participate in complaints processes are vital to their outcomes being perceived as fair and legitimate (Solum, 2004). Active prisoner involvement in the examination of their dispute should be enabled (van der Valk and Rogan, 2020). However, when prisoners are given opportunities to participate it is crucial that their voice does not go unheard, that those listening have the information, interest and power to deal with the complaint(s) raised and where possible, to relieve prisoners of their distress (PRT, 2020). Voices going unheard denies ‘active citizenship’, so prisoners may move to ‘insurgent citizenship’ through physical protests against themselves or the prison authorities (Woltz, 2020). Flawed complaints handling and being ignored can contribute to self-harm and a sense of grievance, leaving prisoners ‘harming themselves to gain some attention, for instance if their applications or complaints are being ignored’ (HMIP, 2020:15; MoJ and HMPPS, 2021). For example, regarding increases in self-harm during 2019–2020, the Independent Monitoring Board (IMB) at HMP Woodhill in England found that ‘many’ suicide prevention documents ‘were associated with men repeatedly not getting answers to often quite straightforward complaints and then self-harming out of frustration’ (IMB, 2020: 10).

Myriad situational, social and structural barriers hinder prisoners’ ability to access and participate in the complaints process (Gill, 2020). These barriers persist despite i) research demonstrating that ‘citizen control’, ‘delegated power’ and ‘partnership’ (Arnstein, 2019: 26) result in procedural fairness, engagement, voice, agency and thus safer prisons and ii) the MoJ and HMPPS complaints policy (2021) stipulating complaints should be supported and that the complaints process should be based on the principles of procedural justice. As such, opportunities to participate in complaints processes and the fairness of complaint *outcomes* form important areas for inquiry.

Moreover, prisoners’ complaints have been found to be constrained in some topics, which is an important consideration for any analysis of complaint categories and prevalence. In California, prisoners lodged complaints relating to ‘universal needs’ including property, medical care and living conditions most frequently – despite disrespect from individual staff members and misconduct ranking highly on prisoners’ lists of issues, prisoners were unlikely to officially file a direct complaint against staff for fear of retribution (Calavita and Jenness, 2013). *What prisoners do not complain about* may therefore be as or more important than what they do submit.

Our critical review methodology combined a substantial literature review with thematic analysis of official data in the public domain, enabling us to conceptualise the research questions and identify gaps in understandings (Barron, 2012: 162). This stage highlighted barriers to prisoners participating in complaints processes. ‘Official’ secondary qualitative and quantitative data were then obtained from recent, publicly available: PPO annual reports and publications (e.g., 2015, 2018, 2020); HMIP (2020) and IMB (2020) annual reports and prisoner surveys; (inter)national prison policies (Council of Europe, 2006, 2017; MoJ/HMPPS, 2021); and domestic reviews (Lammy, 2017; MoJ, 2008). This provided indicative data on complaint frequencies and categories, and prisoners’ perceptions and experiences of the complaints process. This sample facilitated original thematic analysis of secondary data that would be time consuming and difficult to obtain. To our knowledge, these secondary data sources have not previously been analysed to review the complaints process as a mechanism for prisoner participation.

## Case study jurisdiction

**Figure 1.**
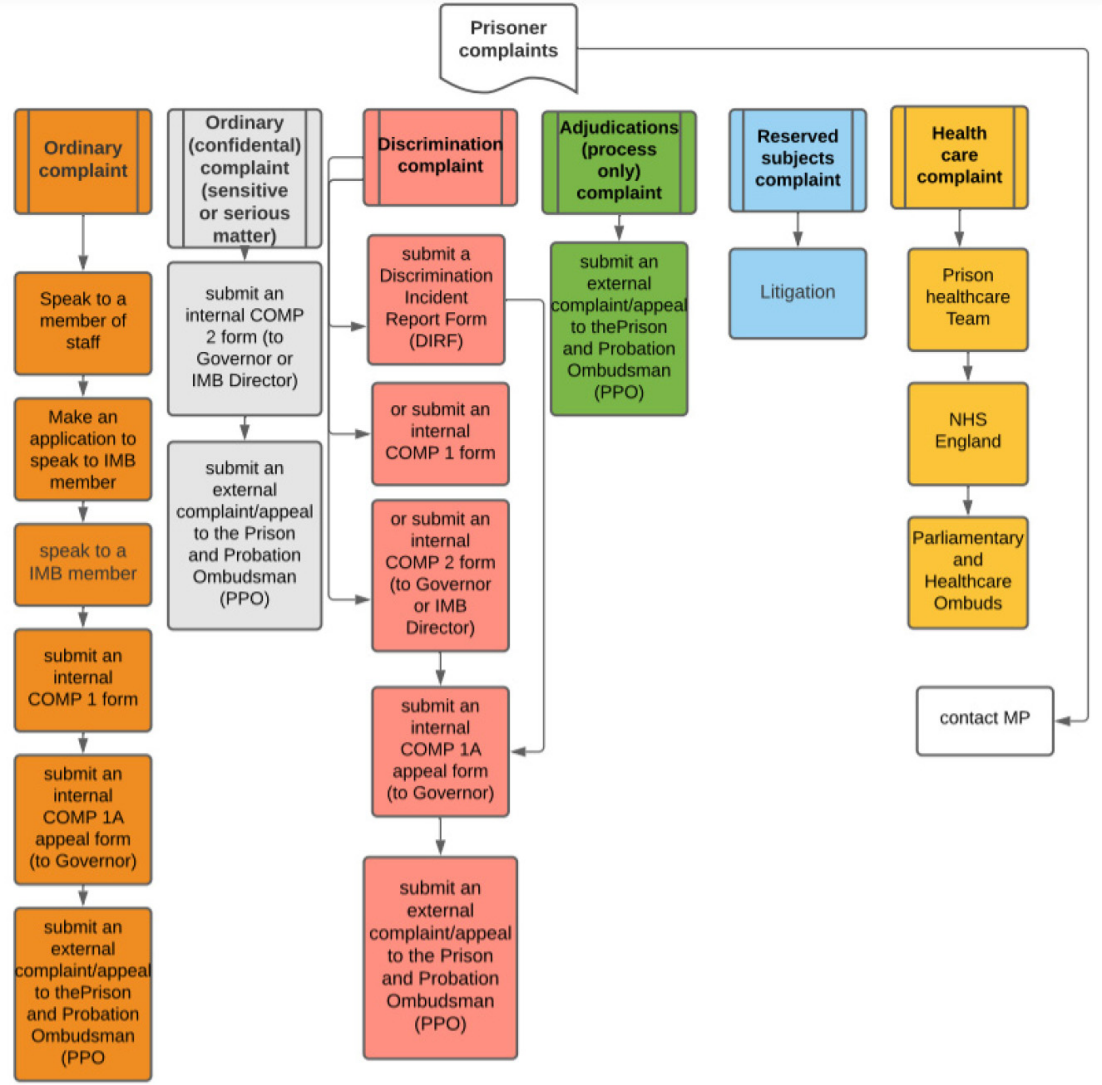
Prisoner complaint process flowchart.

England and Wales has a complex patchwork of complaint mechanisms (see Figure 1). Prisoners can always contact: their MP, who can refer complaints to the Parliamentary Commissioner for Administration (Revolving Doors, 2020); the Prisoners’ Advice Service legal charity, who provide information and advice to prisoners regarding their rights; or a solicitor.^2^ Prisoners under 21 can also contact the Howard League charity's legal helpline.^3^

**Figure 2.**
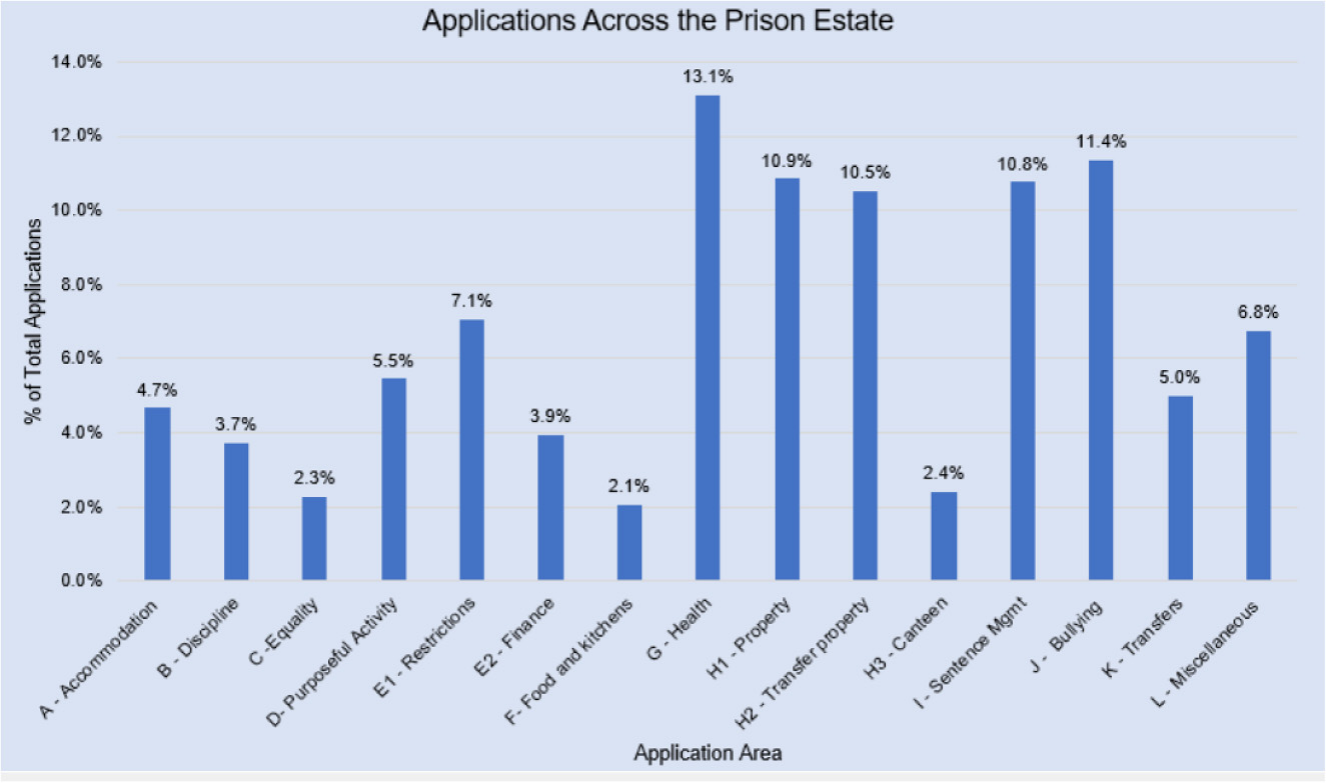
Applications by category. Source: IMB (2020: 37).

**Figure 3.**
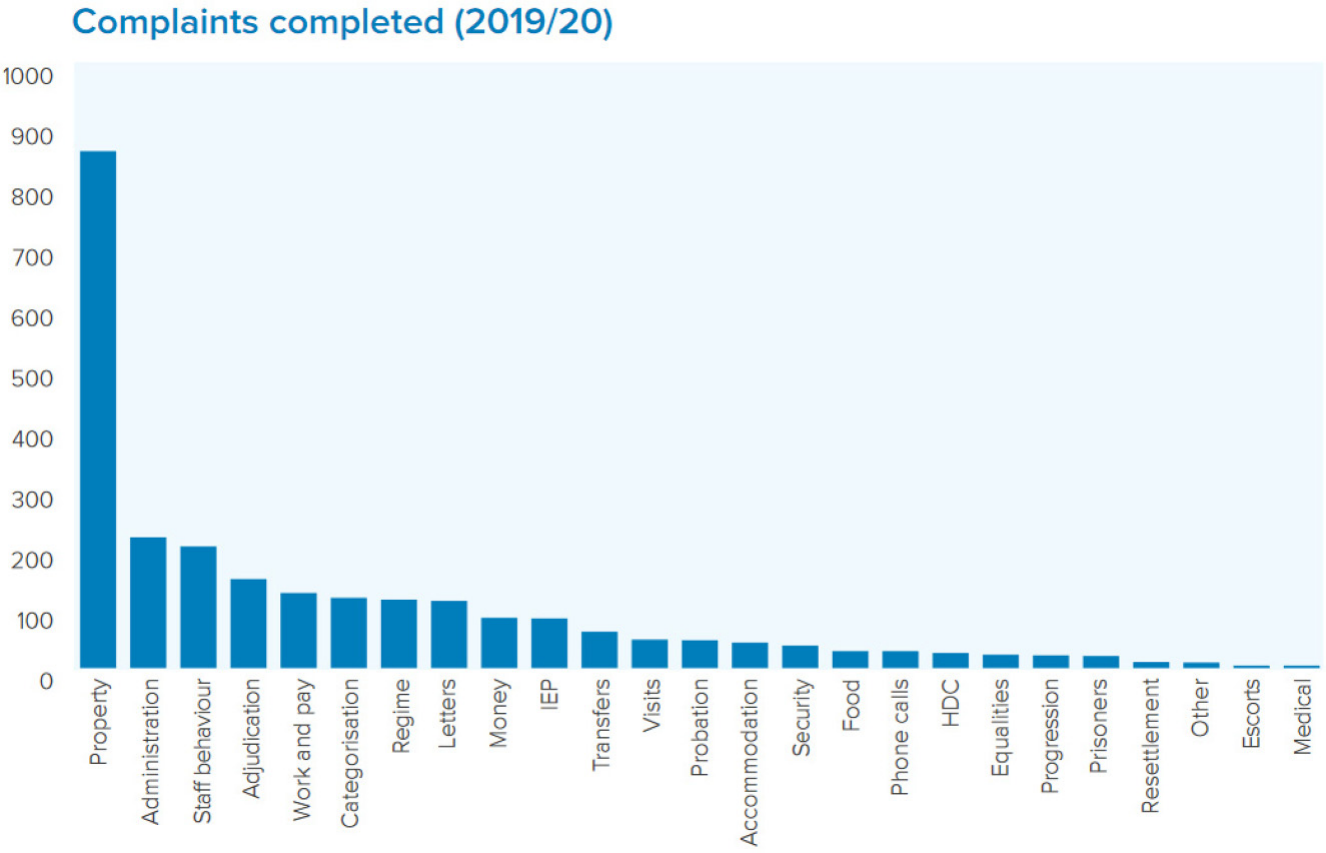
PPO complaints investigated. Note: Figure 3: IEP (Incentives and Earned Privileges) and HDC (Home Detention Curfew). Source: PPO (2020: 18).

Prisoners are firstly expected to raise their concern with a member of prison staff (MoJ and HMPPS, 2021). If this fails, the local IMB must hear any complaint or request which a prisoner wishes to make (known as an application), per the *Prison Act 1952*. The IMB may then attempt to mediate. Their role enables the IMB to follow up individual concerns with prison management and undertake collective analysis of complaints, which purportedly ‘provides a picture of the main issues’ concerning prisoners (IMB, 2020: 34). IMBs comprise volunteers who monitor day-to-day prison life to ‘ensure that prisoners […] are treated fairly and humanely’ and ‘proper standards of care and decency are maintained’ (IMB, 2020). Hearing applications is part of their wider monitoring role. During 2019–20, IMBs received 29,958 applications across the estate (IMB, 2020: 37). The average prison population was around 78,000 (MoJ, 2020) and the number of prisoners cycling through was far higher due to population ‘churn’ and short sentences. Those complaints primarily concerned property (24%) and healthcare (13%), with a significant number relating to sentence management, sentence progression and bullying (Figure 2). However, official complaints data may not be truly representative. ‘Definitional ambiguity’ may result in complaints being selected into different categories than that intended by the complainant (Gavrielides, 2011).

If these steps do not resolve the complaint, prisoners can access the formal internal process. Whilst mediation ‘must be considered as an option to resolve a complaint’ (MoJ and HMPPS, 2021: 7), little information details whether mediation is actually used. Separate mechanisms apply for complaints relating to reserved subjects (allegations against the Governor and litigation against the Prison Service), health care (which is contracted by the NHS) and adjudications (when prisoners have allegedly broken a prison rule). For ‘ordinary’ complaints, prisoners must first complete and submit a COMP1 complaints form to the prison. Forms should be freely available on wings. If dissatisfied with the outcome, a COMP1A internal appeal can be raised to the Governor (MoJ and HMPPS, 2021). ‘Confidential’ complaints relating to sensitive or serious matters can bypass the COMP1 and be made direct to the Governor/ Director or IMB Chair on a COMP2 form, which has no internal appeal mechanism (MoJ and HMPPS, 2021).

Discrimination complaints relating to all nine protected characteristics, for example, race, age, religion, disability, sexual orientation can be raised through three different mechanisms: by ticking the ‘discrimination, harassment or victimisation’ box on a COMP1 or COMP2 form or completing a separate Discrimination Incident Report Form (DIRF). If the discrimination, harassment, or victimisation box is ticked on a COMP1 or COMP2 form, the complaint should also be logged on a DIRF, following the *Equality Act 2010* (MoJ and HMPPS, 2021).

Once internal processes have been exhausted, if a prisoner remains unhappy, they can escalate their complaint to the PPO. The PPO can investigate ‘decisions and actions (including failures or refusals to act) […] relating to the management, supervision, care and treatment of’ prisoners, ranging from use of PAVA incapacitant spray to complaints about missing or damaged property (PPO, 2020: 10). However, few complaints reach the PPO: in 2011, only *3% of the prison population* submitted a complaint appeal following a response to a COMP1A or COMP2 (PPO, 2011: 6).^4^ Vulnerable groups who ‘suffer discrimination and social injustice’ are far less likely to complain. In the community these include young, BAME, disabled and unemployed people (Gill, 2020: 81). Prisoners ‘are generally vulnerable among the most stigmati[s]ed and vulnerable of populations’ (Calavita and Jenness, 2013: 50).

During 2019–20, the PPO received 4686 complaints and completed investigations on 52% (2450) of those submitted complaints. Complaints are not investigated if: ‘the complaint does not raise a substantive issue or if there is no worthwhile outcome’; the complaint is deemed ineligible due to the complainant not going through the relevant internal complaints process (indeed half of ineligible complaints were due to the internal process not being completed); or the complaint was not raised within three months of receiving the final internal outcome (PPO, 2020: 15). Appeals to the PPO should be treated as confidential and prisoners should not be discouraged or prevented from complaining (MoJ and HMPSS, 2020). If still dissatisfied after the PPO's response, prisoners can complain to the Parliamentary and Health Service Ombudsman.^5^

Within the complaints *submitted* to the PPO (2019–20), property was the most common category (28%), followed by staff behaviour (8%) and administration (8%) (PPO, 2020: 15). Of the 2450 complaints investigated*,* 31% of cases were found in favour of the complainant (PPO, 2020: 18).^6^ Property complaints had the highest uphold rate (49%) across all categories (PPO, 2020: 23).

The prevalence of property in IMB and PPO complaints is unsurprising. Loss of property and material issues (e.g., no access to books in cells, living conditions) can have a major effect on prisoners’ sense of dignity (Seneviratne, 2012). ‘Missing or damaged property takes on added meaning’ as ‘personal possessions may be one's only link to the outside world’ and may have increased significance when so many elements of self-identity are stripped away (Calavita and Jenness, 2013: 63; Seneviratne, 2012). Missing property is a ‘perennial problem’ (IMB, 2020: 23) that ‘signals to prisoners that they, like their possessions, have little value’ (Calavita and Jenness, 2013: 65).

HMIP periodically inspects and independently scrutinises conditions and treatment of prisoners. During inspections, prisoners are asked questions including: Is it easy to make a complaint? Are complaints usually dealt with fairly? Have you ever been prevented from making a complaint? (HMIP, 2020: 79). HMIP coordinates the UK's National Preventative Mechanism required by the UN Optional Protocol to the Convention against Torture and other Cruel, Inhuman or Degrading Treatment or Punishment, which the IMB also sits on. As such, complaints are perceived as important and theoretically feed into systemic oversight and torture prevention through this Mechanism (National Preventative Mechanism, 2020; but see Tomczak and McAllister, 2021).

## Barriers

Desk-based analysis highlighted myriad psycho, socio and structural barriers preventing the complaints system from being perceived as participatory, fair and legitimate in process and outcomes. These barriers include culture, demographic differences, accessibility and equitability, timeliness, emotional repression, and bureaucracy. These barriers are important to acknowledge as they serve to undermine prisoner-staff relations, prisoners’ sense of dignity and wellbeing.

### Culture

Organisational culture and power structures make it difficult for prisoners to complain (Seneviratne, 2013). Abilities to complain are socially patterned and ‘affected by power and vulnerability’ (Calavita and Jenness, 2013: 51). Prisons are hierarchical institutions, with ‘unequal distributions of power’: prisoners are in generally disempowered positions, and traditional staff/prisoner relations are built upon hostility and imbalance of power (Calavita and Jenness, 2013: 55). Prisoners with ‘protected’ characteristics are disproportionately disempowered; they feel significantly less likely to be treated with respect, discuss problems with staff, or make a complaint (HMIP, 2020: 118, 120). Structural power relations and social pressures within the prison environment ‘push against the assertion of rights’ (Calavita and Jenness, 2013: 54), potentially leaving prisoners fearful of ‘rock[ing] the boat’ (PPO, 2015: 14).

Traditional hierarchical and discretionary relationships enable prison staff to block complaints and thus control the information communicated to managers and oversight bodies (Woltz, 2020). Prisoner fears of reprisal, which can include for example, downgrading of Incentives and Earned Privileges (IEP) level, loss of prison jobs and detrimental impact on release decisions (PPO, 2015) and being labelled ‘troublemakers’, pose major barriers to complaints (Behan and Kirkham, 2016: 447). The content of complaints is constrained by power relations: prisoners rarely complain about staff behaviour and misconduct for fear of (serious) reprisal from staff (Calavita and Jenness, 2013). Moreover, opportunities to complain about bullying and exploitation from fellow inmates are hampered due to fear of reprisal from other prisoners and prisoner protocols which expect prisoners to remain distanced from staff (De Viggiani, 2012).

In addition, prisoners have ‘widespread mistrust’ and lack of confidence in complaints processes (Padfield, 2018; PPO, 2015: 4). Despite the IMBs’ independent role in monitoring internal complaints and encouraging local resolution, they are perceived by prisoners to be part of scrutiny systems which prisoners have ‘little faith in’ (Justice Committee, 2015: 60). Informal complaints are often met by prison staff apathy, disinterest, lack of empathy, limited information or power to deal with the complaint, which all discourage prisoners from raising issues (PPO, 2015; PRT, 2020). Lack of confidence and trust in complaints processes adds to prisoners’ frustrations, causing ‘problems for the prison’ (Day et al., 2015: 32). Prisoners have also voiced concerns that complaints may be tampered with or destroyed by prison officers (PPO, 2015: 14). A third of adult male prisoners stated they had been ‘prevented from making a complaint when they wanted to’ (HMIP, 2020: 120). Many prison officers are resistant to prisoners becoming active citizens for fear of ‘undermining’ ‘traditional hierarchical power relations’ (Weaver, 2018: 258).

The stability of individual prisons and security categorisation may affect the development of a ‘complaint culture’. Local (Category B) prisons have high numbers of remand and short sentence prisoners hence are more likely to be chaotic, unstable and have fewer resources, thereby further hampering abilities to complain. Interestingly, whilst long term and high security prisoners comprise only 11% of the prison population (PPO, 2020: 15) they are consistently the largest group of complainers to the PPO (PPO, 2015; see also Calavita and Jenness, 2013). Long sentences, the relative stability and focus on order and security in high security prisons may be contributing factors. Where the focus on order and security comes at the expense of ‘interpersonal relations’ prisoners can feel insecurity, disquiet and resentment (Weaver, 2018: 253); potentially resulting in complaints to express frustrations regarding excessive regimes.

### Demographic differences

Prisoners with protected characteristics (including race, age and gender) face discrimination and disproportionate outcomes (Lammy, 2017; MoJ and NOMS, 2008; PRT and ZMT, 2017). Females and younger people are less likely to complain than their older male counterparts within (and outside) prisons (PPO, 2015, 2020). BAME males are significantly more likely than their white counterparts to report consistently poorer experiences with the complaints process and to feel that complaints are not dealt with fairly (HMIP, 2020). BAME male prisoners are also 6% more likely than their white counterparts to have been ‘prevented from making a complaint’ (HMIP, 2020: 120). Discrimination complaints are often among the most troubling received’ but only ‘form a small proportion of all complaints investigated’ (PPO, 2020: 30). An effective complaints process can identify and facilitate mitigation of specific discrimination issues (PRT and ZMT, 2017). However, available PPO data provides only a limited insight into discrimination complaints at either institution level or protected characteristic category. This is an important limitation and area for further consideration.

### Accessibility and equitability

For some prisoners English will not be their first language. In 2021 foreign nationals made up 13% of the prison population (Sturge, 2020). Whilst internal complaint forms are available in nineteen languages to accommodate these prisoners, it is unclear how prisoners obtain these forms. Indeed, prisoners have reported that even standard complaint forms are not always readily available on the wings and must be requested (Behan and Kirkham, 2016). Prisoners who have to request forms or lodge a verbal complaint due to language or literacy barriers may be more reluctant to complain for fear of reprisal or interrogation from prison staff (PPO, 2015).

Nearly half of prisoners have no qualifications and between 20 and 30% have learning difficulties, which decreases awareness of their rights and ability to ask for help to complain (Behan and Kirkham, 2016). Prisoners with learning difficulties, disabilities, language and literacy problems should be given the support they need to submit a complaint (MoJ and HMPPS, 2020). Whilst internal complaints can be taken orally if prisoners have illiteracy or other difficulties, prisoners may not have the confidence to ask for help and may not wish to make this potential vulnerability known (MoJ and HMPSS, 2020: 4, 7).

Complaints may also be limited through lack of awareness of the powers, functions and even existence of complaints processes. Many prisoners simply do not know how to complain and have low awareness and contact with those oversight bodies that are ‘tasked with precisely supporting […] perceptions of safety, respect, fair treatment and the promotion of dignity’ (van der Valk et al. 2021: 17, with reference to Visiting Committees in Ireland which are similar to IMBs). Many prisoners have either extremely limited or zero awareness of the complex complaint appeals process, internally and externally (PPO, 2015), potentially because prisoners are not being provided with information on complaining. Former prisoner Cattermole (2019: 44) was not ‘told a shred’ about how adjudication or complaints processes worked and indeed, staff may withhold this information because they do not want prisoners to complain (Behan and Kirkham, 2016; PPO, 2015).

### Timeliness

Prisoners perceive the internal complaints system to be unnecessarily and deliberately slow (Behan and Kirkham, 2016; PPO, 2015). Complaints are not always dealt with in a timely fashion or within the stipulated timeframes – only 27% of prisoners who had made an internal complaint said it had been dealt with in seven days (HMIP, 2020: 157). Should complaints proceed, it takes the PPO 25 weeks on average to complete their investigation, which is a significant period and likely an impossible delay for the 45% of prisoners serving sentences of 6 months or less (Behan and Kirkham, 2016). This partly explains why only 3% of the prison population submit a complaint to the PPO and why high security prisoners are more likely to complain: they have ‘time and motivation’ (PPO, 2011: 6, 29).

Slow responses may discourage prisoners from lodging complaints and can exacerbate frustrations, potentially resulting in violent acts ‘on wings becoming the ‘norm’’ (PRT, 2020: 42). Delays affect perceptions of fairness and participation: ‘the more often the […] response timeframe is successfully achieved, the more likely the system will be seen as reliable and trustworthy by prisoners’ (MoJ and HMPPS, 2020: 7). The time and speed with which complaints are dealt with is of high importance to prisoners and is linked to dignity: ‘the effective and expeditious processing of prisoners’ queries and requests […] demonstrates respect’ (Hulley et al., 2011: 11).

Prisoners want outcomes to be ‘fair and unambiguous’ (Hulley et al.: 2011: 12). Whilst perceptions of procedural fairness can cushion negative outcomes, (Tyler, 2006) outcomes also matter, and maybe even more so in the prison setting where power imbalances are known to dominate (Jenness and Calavita, 2018). If the process continuously delivers unfavourable outcomes or fails to promote change the ‘ability of the procedure to cushion the outcome’ may be undermined (Tyler, 2006: 107). Prisoners will not engage if they perceive their efforts as a ‘waste of time’ (PPO, 2015: 4; van der Valk and Rogan, 2020).

Many prisoners view the complaints system to be ‘a waste of time’ due to its perceived inability to initiate change (PPO, 2015: 4; van der Valk and Rogan, 2020). A reoccurring theme amongst prisoners is that there is no point lodging a complaint - complaints will either be ignored or not taken seriously, hence nothing happens as a result. In the words of one former prisoner: ‘what's the point in the whole complaint process if nothing's gonna happen’? (Revolving Doors, 2020: 16). Prisoners want their concerns heard and taken seriously: being ‘fobbed off’ or ignored could have ‘considerable implications for prisoners’ futures’ and ‘levels of institutional order’ (Hulley et al., 2011: 12, 20).

Whilst 31% of complaints investigated by the PPO in 2019–2020 were upheld in favour of the complainant (PPO, 2020) there is little collated data demonstrating the number of internal complaints made or upheld. However, PRT and ZMT (2017) established that only 1% of ‘alleged discrimination by an officer’ complaints lodged by prisoners internally were upheld (PRT and ZMT, 2017: vii) compared to 76% upheld rate for staff ‘alleged discrimination by a prisoner’ complaints. This is another important limitation and area for further consideration.

### Emotional repression

A key coping mechanism is ‘emotional repression’ whereby prisoners must act like a ‘real’ man by displaying ‘physical, psychological, and emotional strength’ (Maguire, 2019: 6) and not show any signs of weakness, vulnerability, or emotion (De Viggiani, 2012). Adopting a victim status can form an invitation to be bullied or exploited (De Viggiani, 2012). Hegemonic prison masculinity may affect whether a prisoner is willing to see themselves as a victim or not. Prisoners may reject victim status and deny injury as an ‘ethic of survival’ (Bumiller, 1987). In some cultures endurance is positively valued: an ‘ethic of survival’ has been found amongst black males as a mean of preserving self-respect amidst racial discrimination or victimisation (Bumiller, 1987). Standing up for oneself through acts of aggression or violence ‘is one of the defining characteristics of hegemonic prison masculinity’ (Maguire, 2019: 505). Younger males are more likely to use violence or aggression to ‘voice’ frustrations whilst older male prisoners tend to use other characteristics of hegemonic prison masculinity including ‘stoicism’, not being an ‘informer’, self-sustainability, showing and commanding ‘respect’ to, and from, fellow prisoners and staff (Maguire, 2019: 506). This may explain why younger prisoners tend not to complain and the majority of complaints received by the PPO are from males aged 30 years and above (PPO, 2011: 8).

Furthermore, many prisoners feel they ‘deserve to be ill-treated’ as a consequence of their rule breaking and subsequent incarceration (PPO, 2015: 14). This self-blame results in prisoners believing that they do not have the right to complain, with any issues encountered being part of their punishment. Self-blame along with vulnerability, stigmatisation or being located in a ‘vulnerable social location’ (such as prison) can led to a sense of disentitlement and an unwillingness or inability to engage in complaints processes (Calavita and Jenness, 2013: 51).

### Bureaucratic barriers

Bureaucratic barriers also appear to constrain complaining. As noted above, nearly two-thirds of complaints escalated to the PPO were deemed not eligible for investigation, half of those due to the internal complaints process not being completed (PPO, 2020). Many prisoners simply do not know or understand the different stages that must be completed before complaints can be escalated to the PPO (PPO, 2020). Such extensive, systemic misunderstanding raises fundamental questions of participation, accessibility and equitability. Moreover, the lack of data available regarding the number of internal complaints made or upheld and the categories and frequencies of these complaints leaves individual prisons and prison staff unaccountable for the outcomes of internal complaints unless complaints are escalated to the PPO. This serves to obfuscate internal complaints and compound the influence of staff discretion which could result in ‘denial of voice, and the failure to wield authority in a fair, unbiased and neutral manner’ (Jackson et al., 2010: 7).

More specifically, there is a lack of external scrutiny in regard to discrimination complaints. Under the *Equality Act 2010*, prisons have a duty to eliminate unlawful discrimination, harassment and victimisation. ‘Ensuring complaints about discrimination are investigated promptly and effectively’ is ‘one important way in which HMPPS can fulfil its responsibilities’ for equality (PPO, 2018: 1). Prisons are encouraged to expose the DIRF review process ‘to outside scrutiny, including the way in which complaints about discrimination are handled’ but it is not a mandatory requirement (Lammy, 2017: 45). Discrimination complaints only ‘form a small proportion’ of PPO investigations (PPO, 2020: 30). PPO scrutiny indicates that ‘all too often discrimination complaints are not investigated promptly, that the staff who investigate them often lack the training and confidence to address equalities issues effectively, and that prisons often fail to collect the equalities data needed to carry out a meaningful investigation’ (Prisons and Probation Ombudsman, 2018: 1). Furthermore, HMIP (2020: 41) noted that ‘many prisons had poor systems to handle complaints about discrimination’ which serves to undermine ‘prisoner confidence in the process’.

There is also limited visibility of complaints regarding the IEP system. The IEP system was an arbitrary system perceived by many prisoners to be deeply unfair. The IEP system contributed to the 1990 prison disturbances (PRT, 2014; Woolf, 1991) and was one of the twelve recommendations made in Woolf's independent inquiry (Woolf, 1991). Initially overhauled in 1995, a subsequent revision of the IEP system in 2013 led to ‘a strong sense of grievance and injustice’ (PRT, 2014: 1), undermining ‘trust […] [,] threaten[ing] […] legitimacy’ and creating ‘instability’ (Pratt and Garton Grimwood, 2014: 3). The system eroded prisoners’ perceptions of fairness and decency of prison regimes (Day et al., 2015), yet there is no accessible data on the frequency of IEP complaints at either individual prison or prison estate level nor does the PPO provide data beyond the total number of IEP complaints.

All these barriers constrain the complaints process’ utility as a mechanism of prisoner participation that could foster fairness, legitimacy and dignity. Prisoners are not always provided with, or aware of, the information enabling them to complain (van der Valk et al., 2021). If prisoners are meet with apathy, merely told to ‘ignore it’ (PRT, 2020: 26) and feel that there is no point in lodging complaints because nothing ever changes, this suggests that, at best, prisoner participation through complaints is limited to the ‘placation’ rung and at worst, the ‘manipulation’/‘no power’ rung on the ‘ladder of citizen participation’ (Arnstein, 2019: 26).

## Discussion

Although we are a very long way from a system that enables meaningful prisoner participation, an ‘increasing number of academics have begun to advocate for the broader possibilities of restorative justice in prisons within adjudications, prisoner councils and other ways of working’ (including complaints) (Calkin, 2021: 94). Whilst little researched, several scholars (see Butler and Maruna, 2016; Calkin, 2021; Dhami et al. 2009; Edgar and Newell, 2006) argue that restorative justice/practice (within prisons) can be an effective tool in dealing with bullying, victimisation, complaints and adjudications, improving prisoner experience and making prisons more democratic, humane and less violent. Adopting a ‘complaint culture’ modelled around restorative justice/practice and procedural fairness goals - inclusivity (participation and decision-making), bi-directional dialogue, voice, fairness and agency – would provide an alternative to the current adversarial model with binary outcomes and could ‘offer a viable redress’ to legitimacy issues, improve poor staff-prisoner relations, enhance prisoner well-being and support civic engagement (Beijersbergen et al., 2015; Butler and Maruna, 2016: 126). As such, restorative justice could better facilitate prisoner voice and thus help meet the potential of complaints to be participatory, fair and inclusive in practice and forming the ‘lever by which to shift conditions’ (Mika and Thomas, 1988: 57). Implementation barriers include: lack of awareness, training, funding, and organisational culture (Banwell-Moore, 2019; Gavrielides, 2011).

A recent study examined the implementation of restorative practice within three CAT C and D prisons (holding ‘a complex population’).^7^ The prisons were selected as they ‘demonstrate[d] outwardly a commitment to RP [restorative practice] and are indicative of good cultures, according to recent MoJ data’ (Calkin, 2021: 92). Calkin (2021: 107) determined that ‘employing restorative practice techniques can prevent and de-escalate conflict, manage challenges and deliver a ‘culture of fairness’. Several other prisons have implemented ‘restorative adjudications’ processes (Gavrielides, 2011): HMP Brixton; Bullingdon and Grendon. Whilst there has been no evaluation of ‘restorative adjudications’ processes (only a mapping exercise, see Gavrielides, 2011), restorative techniques can be ‘appropriate means of handling […] conflicts and tensions’ within prisons (Barabas et al., 2012: 39). However, encouraging a ‘complaint culture’ requires that the ‘institutional and cultural dynamics’ of the prison be challenged (Behan and Kirkham, 2016).

## Conclusion: Towards participatory complaints mechanisms

This article has examined how the prison complaints system in England and Wales works in principle and practice, and the process(es) that prisoners have to negotiate if they wish to complain. Whilst prisoners’ actions have directly shaped complaints mechanisms available today, the complaints system in England and Wales is convoluted and lengthy, and many prisoners are unable or unwilling to navigate it for various structural and situational reasons - as detailed above. This critical review of literature and secondary data analysis on the complaints system, categories and frequencies has demonstrated numerous, significant barriers to complaints forming a mechanism that facilitates prisoner participation and meeting their potential to stimulate inclusive prisoner voice, agency and rights protection, in turn averting self-harm and violence, and facilitating systemic improvements. Moreover, this paper has illustrated that cognisance of the levels and management of complaints within the prison service is limited. As complaints must pass through internal mechanisms before being escalated to the PPO, this is an important limitation.

The current complaints system is meant to offer prisoners opportunities to have active voice and enable trends in complaints to be identified in order to ‘proactively initiate change’ (MoJ and HMPPS, 2020: 14). Whilst complaints mechanisms hold clear potential to enhance prison legitimacy, facilitate prisoner engagement and agency, and improve wellbeing and safety, the extent to which prisoners’ voices can initiate prison reform at local or national level depends very much on the ‘information channels’ available, prisoner demographics, the organisational culture, external scrutiny and the level of visibility of complaint categories and frequencies. Internal complaints processes and prisoners’ lived experiences of the complaints system require further exploration to illustrate how to better foster a restorative ‘complaint culture’ that facilitates prisoner voice in order to be participatory, inclusive and fair.

## Notes

1. Some jurisdictions (excluding the UK) have ‘own-initiative powers of investigation’ which enable Ombud institutions to investigate systemic injustices. In Northern Ireland ‘own initiative powers of investigation’ can be launched where the ombud has a “reasonable suspicion” that “there is systemic maladministration” or “systemic injustice”’ (Gill, 2020: 79).

2.
http://www.prisonersadvice.org.uk/

3.
https://howardleague.org/legal-work/advice-line/

4. In the community, ‘27% of people who complain to a service provider and remain dissatisfied’ escalate their complaint to ombud institutions (Gill, 2020: 81).

5.
https://www.ppo.gov.uk/investigations/make-complaint/appealppodecision/

6. Comparative Ombud complaint upheld rates vary widely, but prisoner rates appear low: Financial Ombudsman Service (2021) 38%; Parliamentary and Health Care Ombudsman (2021) NHS complaints 48%; Local Government and Social Care Ombudsman (2021) 72%.

7. Cat C prisons are for prisoners who cannot be trusted in open prison but who are unlikely to attempt escape. Cat D prisons are ‘open’ prisons.
